# Hippocampal Sclerosis After Legionnaires’ Disease: A Case Report

**DOI:** 10.7759/cureus.75773

**Published:** 2024-12-15

**Authors:** Atsuko Ikenouchi, Satoru Ide, Gaku Hayasaki, Hiroaki Adachi, Reiji Yoshimura

**Affiliations:** 1 Department of Psychiatry, University of Occupational and Environmental Health, Japan, Kitakyushu, JPN; 2 Medical Center for Dementia, Hospital of the University of Occupational and Environmental Health, Japan, Kitakyushu, JPN; 3 Department of Radiology, University of Occupational and Environmental Health, Japan, Kitakyushu, JPN; 4 Department of Neurology, University of Occupational and Environmental Health, Japan, Kitakyushu, JPN

**Keywords:** acute symptomatic seizure, cognitive impairment, hippocampal sclerosis, legionnaires’ disease, neurological symptoms

## Abstract

Legionnaires’ disease is a form of atypical pneumonia that can present with neurological symptoms, such as headaches, seizures, and focal neurological abnormalities. We report the case of a male patient who developed impaired consciousness and recurrent seizures following pneumonia caused by *Legionella*. The patient received antibiotics and antiepileptic treatment and was discharged on hospital day 56. He was diagnosed with hippocampal sclerosis 10 months later. To our knowledge, this is the first reported case of hippocampal sclerosis following Legionnaires’ disease globally. Clinicians should be aware of the risk of hippocampal sclerosis after Legionnaires’ disease.

## Introduction

*Legionella* spp. are present in various natural and artificial water environments, including cooling towers, water supply systems, and hot water environments and are a common cause of community-acquired pneumonia. Pneumonia and systemic infections caused by *Legionella* are known as Legionnaires’ disease. Legionnaires’ disease is likely to occur during the summer and early fall, and its incidence increases annually [1].

Legionnaires’ disease is an atypical pneumonia clinically similar to pneumococcal pneumonia and pneumonia caused by other bacteria. Symptoms often include fever, cough, chills, dyspnea, myalgia, diarrhea, and chest pain, and neurological abnormalities occur in 38-53% of patients [1]. The neurological symptoms of *Legionella *infection include disorientation (58%), headache (52.4%), and somnolence (39.7%), whereas memory impairment (1.6%) and seizures (1.5%) are uncommon [2]. These neurological symptoms are often a result of infectious encephalopathy.

There have been no reported cases of hippocampal sclerosis after Legionnaires’ disease. We report the first case of hippocampal sclerosis following pneumonia due to *Legionella*.

## Case presentation

A 69-year-old man presented with memory impairment at the Medical Center for Dementia, Hospital of University of Occupational and Environmental Health, Japan. He had no history of epilepsy or dementia but had been diagnosed with Legionnaires’ disease 10 months earlier.

**Figure 1:**
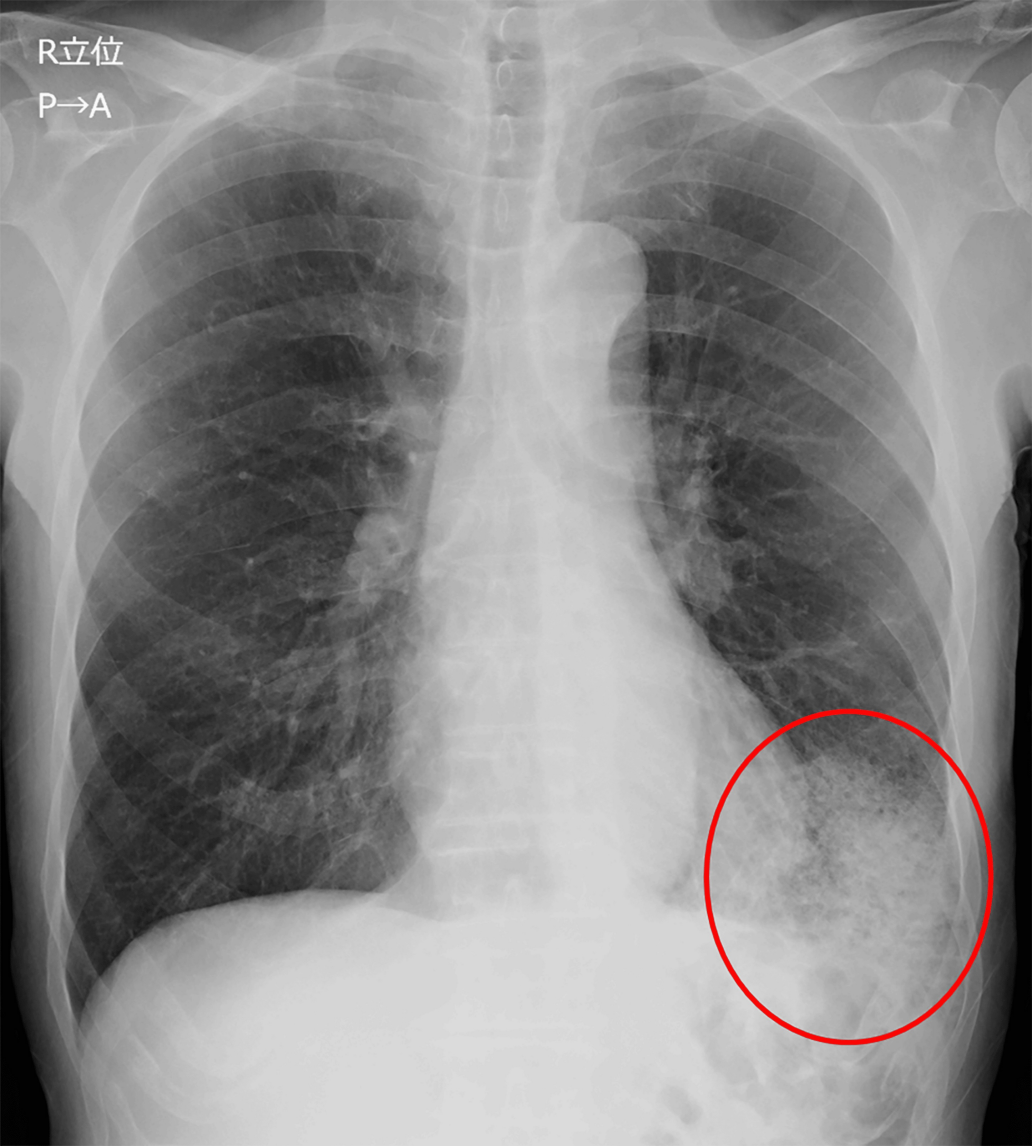
Chest radiography on admission Chest radiography reveals a consolidation in the left lower lung field, suggestive of pneumonia (circle).

In October X-1, the patient developed chills and a fever in the 37°C range, followed by a gradual onset of numbness in the right lower limb. The patient visited the Department of Internal Medicine the following day. The patient was alert, with a respiratory status showing a SpO_2_ of 98% on room air. Blood tests showed a white blood cell count of 10,100/μL (reference range: 3300-8600/μL) and a C-reactive protein (CRP) level of 20.97 mg/dL (reference range: 0.0-0.14 mg/dL). On the chest X-ray, an infiltrative shadow was observed in the left lower lung field (Figure 1).

**Figure 2:**
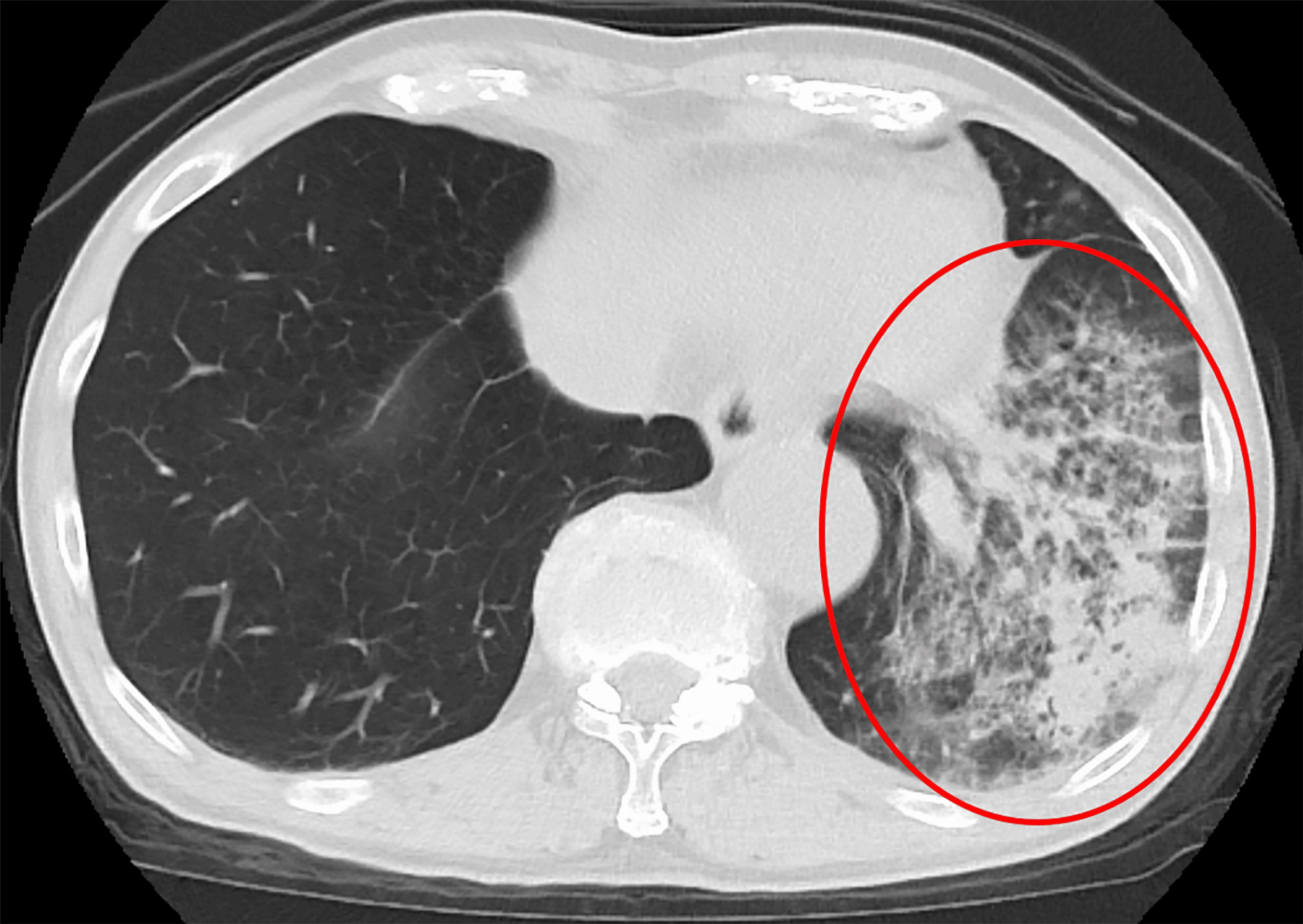
Chest CT on admission The chest CT scan taken shows consolidation in the left lower lobe (circle), consistent with findings of pneumonia.

Plain chest computed tomography with a standard radiation dose confirmed extensive consolidation in the left lower lobe (Figure 2).

He was diagnosed with pneumonia and admitted to hospital on the same day. Blood, sputum, and urine cultures were performed to identify the causative agent, but both urinary *Legionella* and pneumococcal antigens were negative. Sulbactam sodium plus ampicillin sodium (SBT/ABPC) relieved his fever.

**Figure 3:**
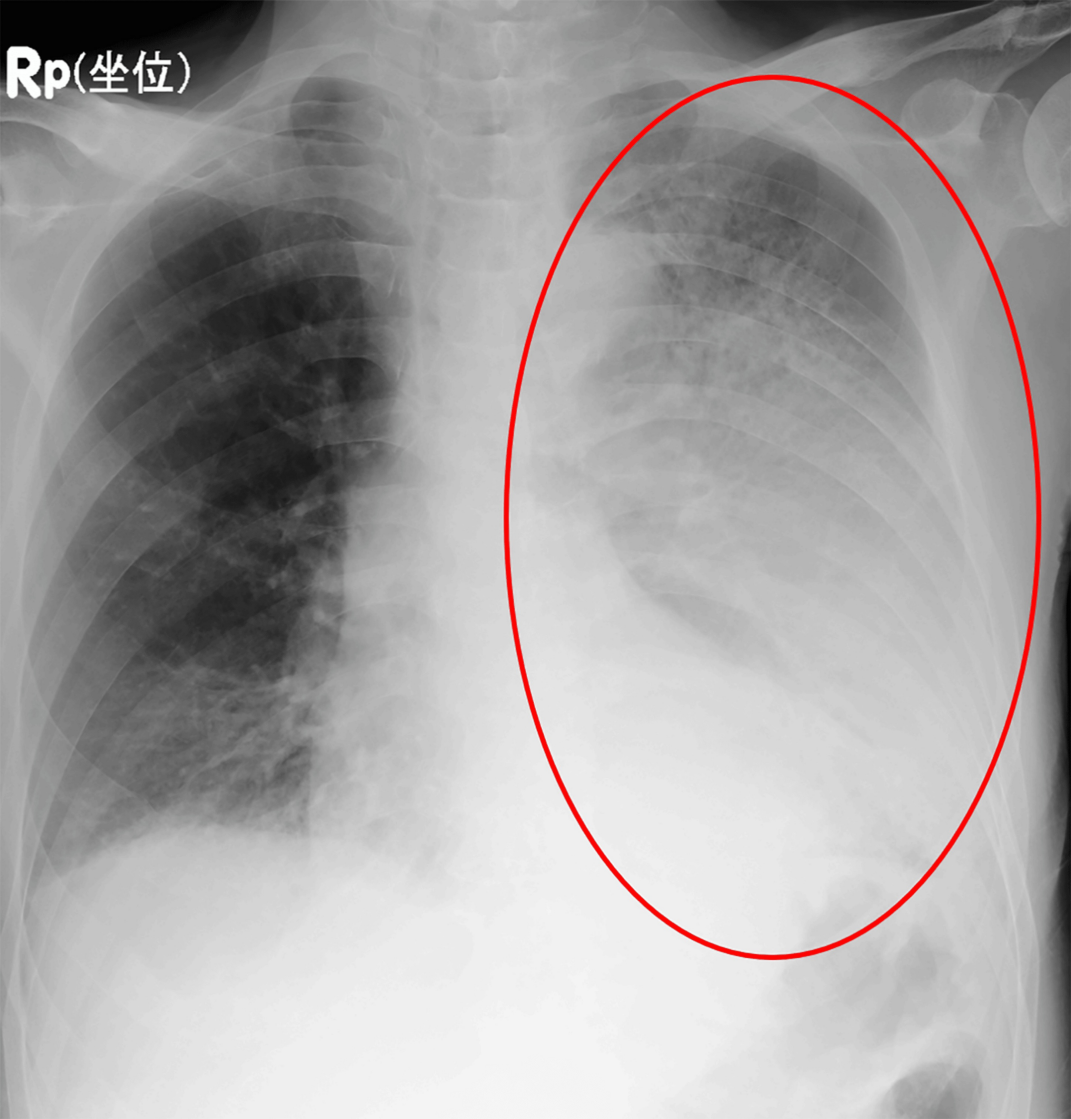
Chest radiography on hospital day three Chest radiography shows enlargement of the infiltrative opacity in the left lung field (circle).

On hospital day three, he developed a fever at approximately 39°C and breathlessness, and chest radiography showed enlargement of the infiltrative opacity in the left lung field (Figure 3).

**Figure 4:**
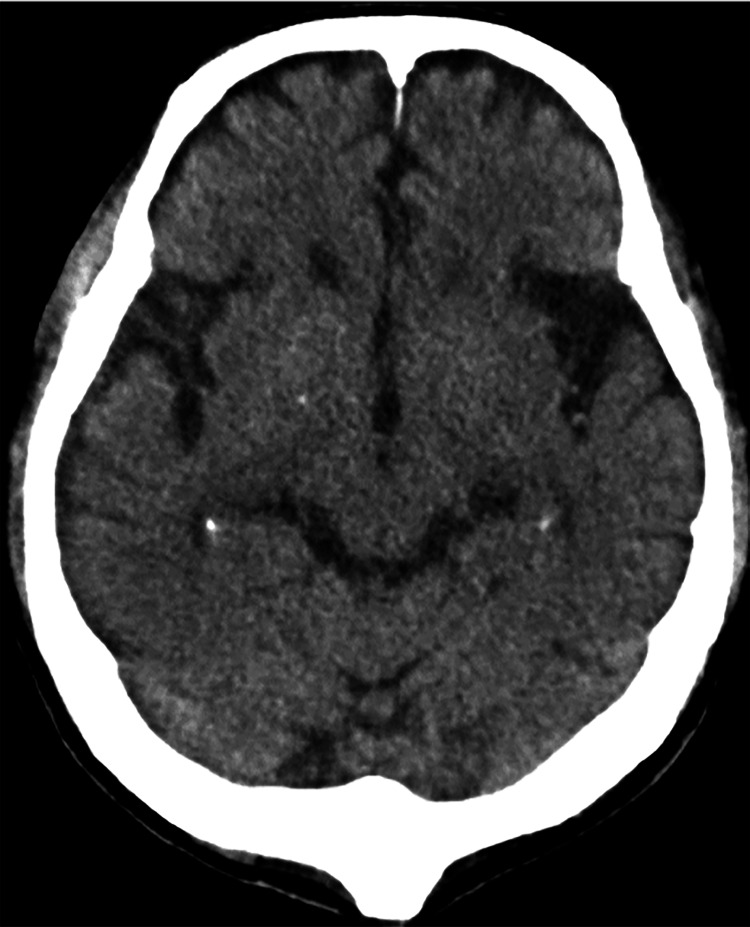
Axial head CT for acute symptomatic seizures Head CT reveals no acute lesions such as intracranial hemorrhage. No evidence of atrophy is apparent.

His antibiotic was changed from SBT/ABPC to tazobactam plus piperacillin (TAZ/PIPC). On hospital day five, the fever persisted, and hallucinations appeared, which were thought to be due to delirium. Levofloxacin (LVFX) was added to treat atypical pneumonia. On hospital day six, despite oxygen administration at 7 L/min via a reservoir mask, the patient could barely maintain a PaO_2_ of 55.5 Torr. Consequently, the patient was transferred to the Department of Respiratory Medicine. On hospital day seven, repeated sputum culture detected staphylococci, and vancomycin (VCM) and doripenem were administered. On hospital day eight, he experienced a decline in consciousness and seizures involving the neck, lower jaw, and right upper and lower limbs. Although intravenous administration of diazepam (5 mg) temporarily controlled the seizures, they recurred. Blood tests revealed a white blood cell count of 23,800/μL and a CRP level of 41.87 mg/dL. Plain head CT revealed no acute lesions, such as intracranial hemorrhage. Scattered, small, nonspecific calcifications were observed in both hippocampi, although there was no evidence of atrophy (Figure 4).

Acute symptomatic seizure (ASS) was suspected due to his deteriorating clinical condition, likely aggravated by antibiotic treatment, specifically doripenem. Doripenem was discontinued because extended-spectrum beta-lactamases were not detected in any cultures. The patient was a carrier of methicillin-resistant *Staphylococcus aureus*; therefore, VCM treatment was continued. Fosphenytoin (initial dose: 22.5 mg/kg; maintenance dose: 7.5 mg/kg) and levetiracetam (1,000 mg) were administered due to frequent recurrent seizures.

**Figure 5:**
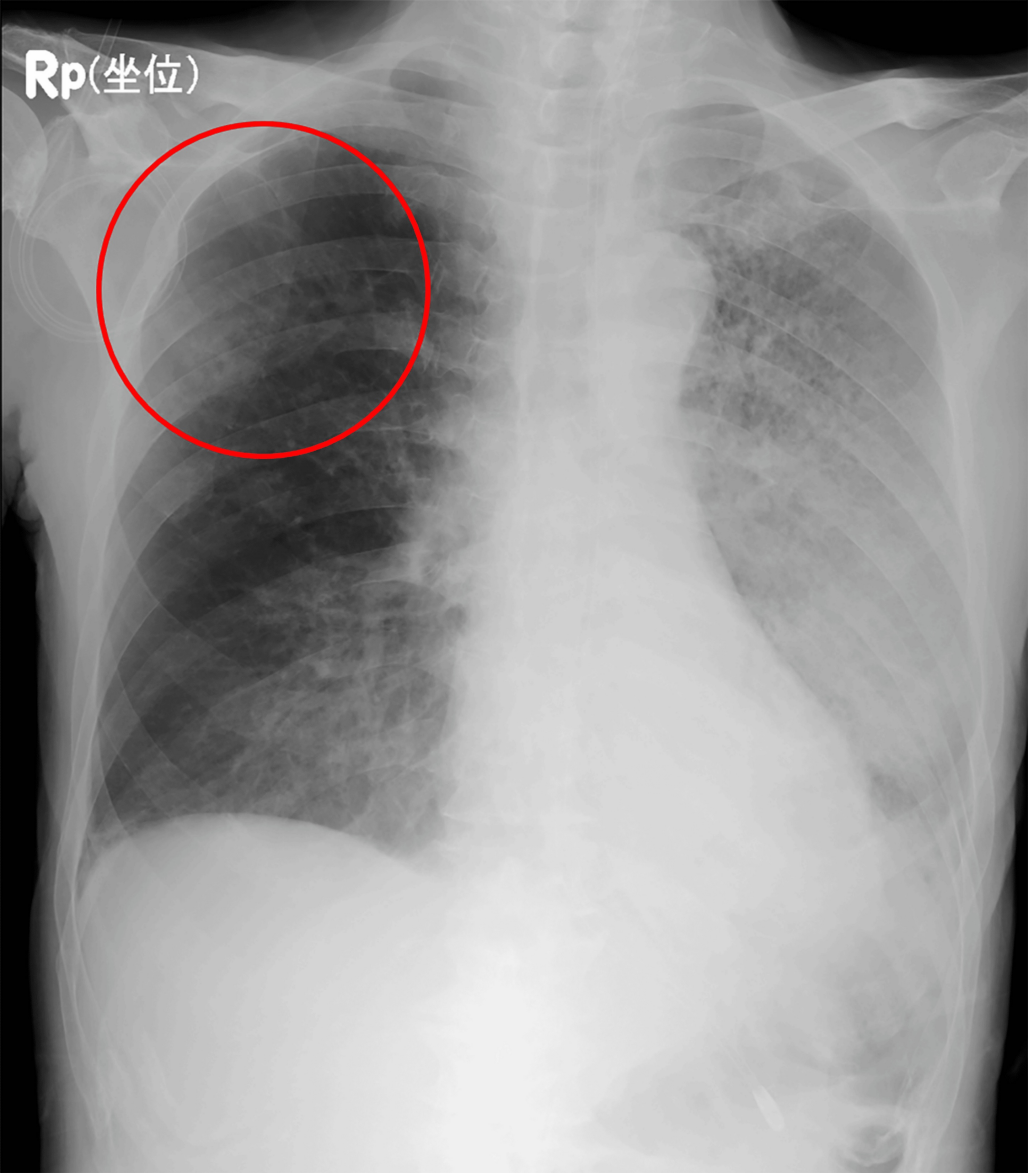
Chest radiography on hospital day 14 Chest radiography shows a new infiltrative opacity in the right upper lung field (circle).

On hospital day nine, a test for *Legionella* DNA (serotype unknown) using loop-mediated isothermal amplification (LAMP) was positive. LVFX and TAZ/PIPC were continued treatment for aspiration pneumonia as the patient remained unconscious. VCM for methicillin-resistant *S. aureus* was discontinued due to the lack of phagocytosis images. On hospital day 12, his fever subsided to the 37°C range, and consciousness was restored. Laboratory results showed improvement, with a white blood cell count of 9,200/μL and a CRP level of 10.32 mg/dL. Fosphenytoin was discontinued, and levetiracetam (1,000 mg) was continued. On hospital day 14, his white blood cell count was elevated again. He exhibited a fever at approximately 38°C and was observed talking to himself. A new infiltrate shadow was revealed in the right upper lung field on chest radiography (Figure 5).

**Figure 6:**
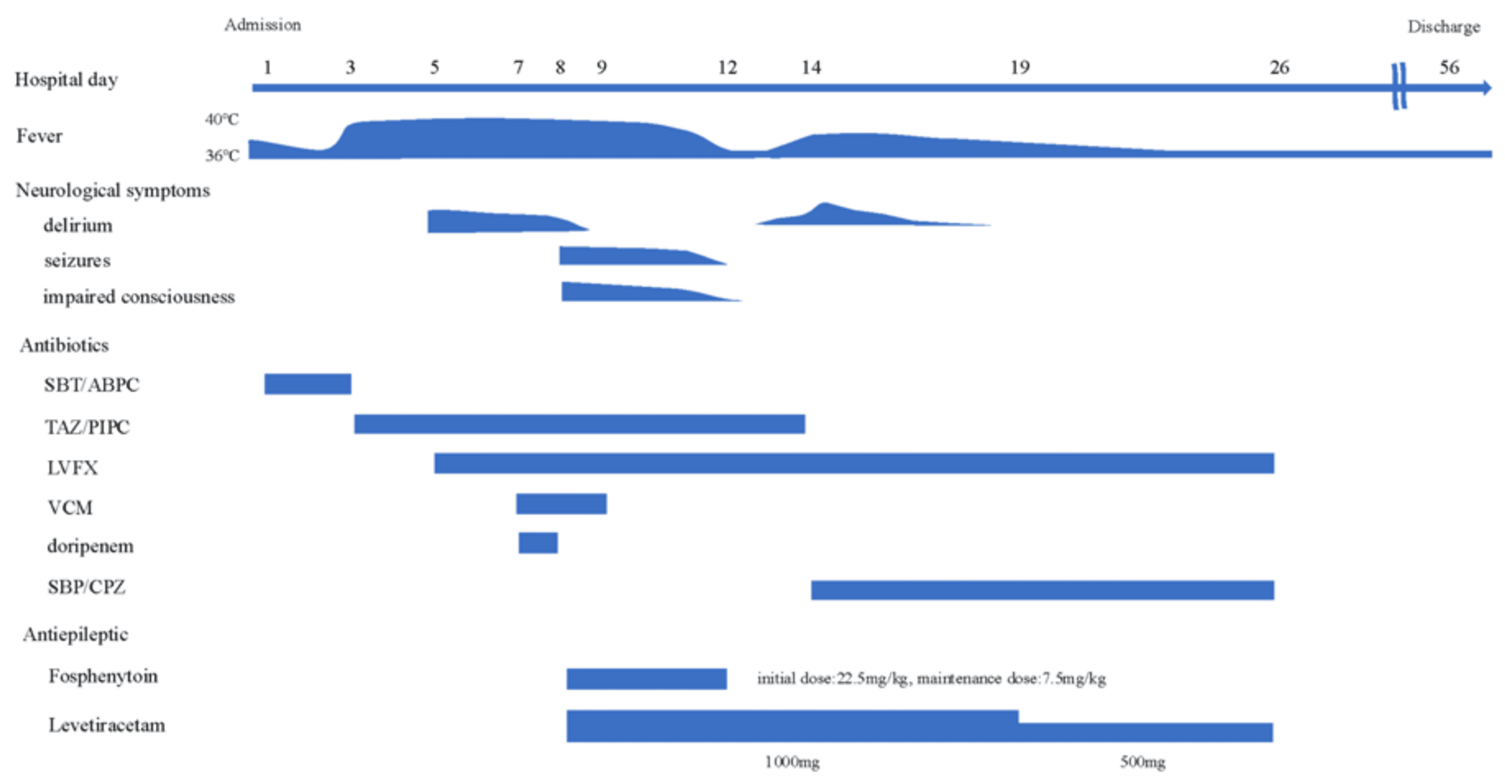
Clinical course during hospitalization SBT/ABPC: sulbactam sodium plus ampicillin sodium, TAZ/PIPC: tazobactam plus piperacillin, LVFX: levofloxacin, VCM: vancomycin, SBP/CPZ: sulbactam plus cefoperazone

TAZ/PIPC was changed to sulbactam plus cefoperazone (SBP/CPZ) due to a skin rash. On hospital day 19, the levetiracetam dose was reduced to 500 mg and administered in two divided doses due to the absence of recurrent seizures. On hospital day 26, his overall clinical condition improved, with no seizure recurrences and focal neurologic deficits; LVFX and SBP/CPZ were discontinued, along with levetiracetam. The patient was followed up without preventative medication, as seizure symptoms did not recur. He underwent rehabilitation for the disuse syndrome and was discharged on hospital day 56 (Figure 6).

**Figure 7:**
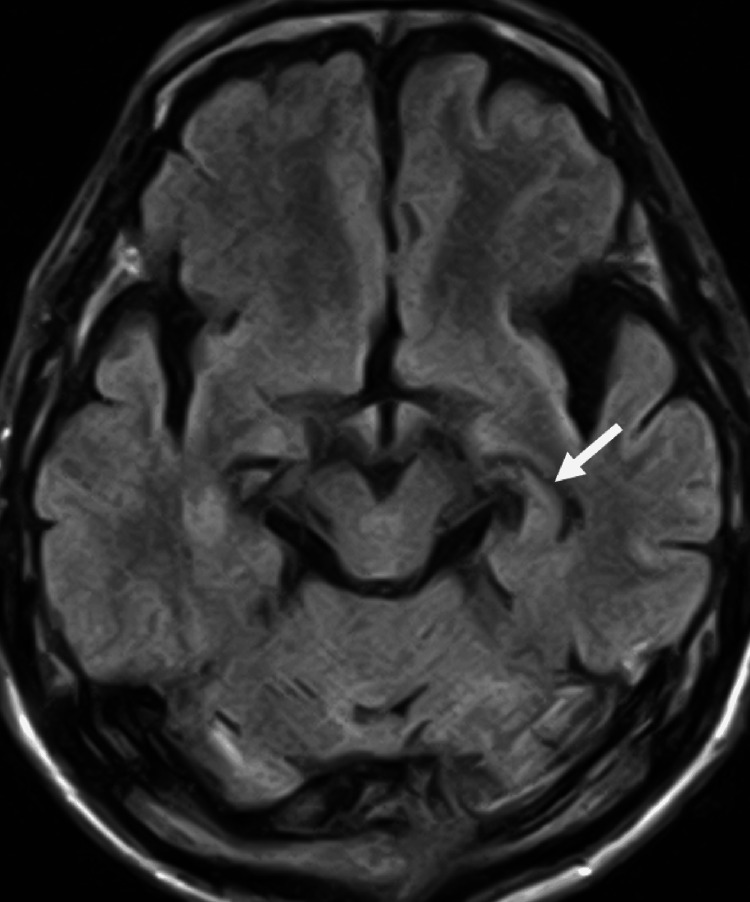
Axial head MRI (fluid-attenuated inversion recovery imaging) at the time of detection of memory impairment The left hippocampus appears mildly atrophic compared with the contralateral side (arrow) although optimal protocols for evaluating the hippocampus were not used.

In December X-1, he began mumbling noticeably on the phone and sometimes failed to respond when called while watching TV. In February X, he visited a neurosurgeon because he could not recall the letters, names of people, or objects. He was suspected to have mild cognitive impairment, with scores of 17/30 on the Hasegawa’s Dementia Scale-Revised and 23/30 on the Mini-Mental State Examination. Optimal protocols for evaluating the hippocampus were not used during head magnetic resonance imaging (MRI), but the left hippocampus appeared mildly atrophic relative to the right, with dilatation of the left temporal horn, in the fluid-attenuated inversion recovery images (Figure 7).

He occasionally became disoriented at work. In July X, he was involved in an accident; he hit several cars while driving, but did not process the accident and only became aware of it when a police officer visited his home. He remembered contact with the first car and its color but could not recall what happened next.

**Figure 8:**
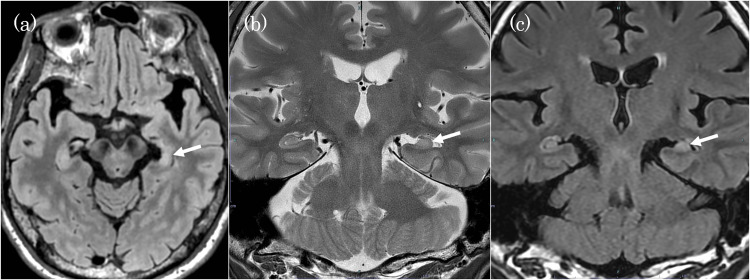
Head MRI at six months after the detection of memory impairment (a) Axial fluid-attenuated inversion recovery image. (b) Coronal T2-weighted image. (c) Coronal fluid-attenuated inversion recovery image. The left hippocampus shows atrophy (arrow in a), loss of hippocampal striation (arrow in b), and an elevated T2 signal (arrow in c), which are consistent with hippocampal sclerosis. Specific changes in C1 or C3 regions associated with hypoxic-ischemic encephalopathy could not be evaluated.

In August X, he was suspected of having dementia and visited the Medical Center for Dementia. On examination, he had an intact mental status and no focal neurologic deficits. He had a seizure in the hospital, becoming unresponsive with unnatural hand movements. Neuropsychological testing yielded the following scores: Hasegawa’s Dementia Scale-Revised, 16/30; Mini-Mental State Examination, 25/30; Clock Drawing Test (Freedman method), 12/15; Montreal Cognitive Assessment, 18/30; Frontal Assessment Battery, 5/18; and Cognistat Five, 6A. Disorientation, visuospatial cognition, organization, and language function were relatively well retained, but memory and frontal lobe function showed marked impairment. The Japanese Adult Reading Test predicted an overall test intelligence quotient (IQ) of 94, a verbal IQ of 93, and a motor IQ of 95. The Wechsler Adult Intelligence Scale, 4th edition (WAIS-IV) showed a full-scale IQ of 59, verbal comprehension index of 67, perceptual reasoning index of 71, working memory index of 67, and processing speed index of 66. Hematological and biochemical tests, including endocrine and metabolic panels, showed no abnormalities. Holter electrocardiography showed no finding that could cause a transient loss of consciousness or syncope. Head MRI revealed left-dominant medial temporal lobe atrophy with a hyperintense T2 signal and an obscured laminar hippocampal structure, suggesting hippocampal sclerosis (Figure 8).

**Figure 9:**
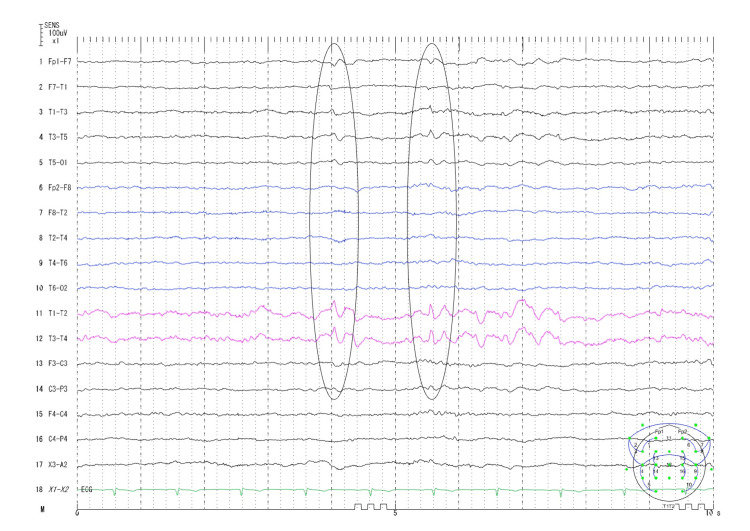
Interictal electroencephalographic findings Left temporal spikes and sharp waves with secondary involvement of the left frontal region were noted during sleep in bipolar montage (circle).

Electroencephalography (EEG) revealed persistent sharp waves in the left temporal region, and mesial temporal lobe epilepsy secondary to hippocampal sclerosis was diagnosed (Figure 9).

Lacosamide was initiated at 100 mg and titrated to 200 mg. However, as seizures persisted, levetiracetam was added at a dose of 1,000 mg. Both medications were administered in two divided doses per day. Subsequently, the seizures resolved.

## Discussion

This case describes hippocampal sclerosis diagnosed 10 months after the initial presentation of neurological symptoms following a *Legionella *infection, including impaired consciousness and convulsions. The initial blood, sputum, and urine cultures and urinary *Legionella* antigen tests were negative on admission, but the patient was diagnosed with Legionnaires’ disease on the hospital day nine based on a positive result for *Legionella* DNA using the LAMP method and clinical symptoms. *Legionella* bacteria are difficult to detect as they do not grow well on standard culture plates. Urinary antigen tests have 99% specificity for *Legionella pneumophila* serogroup 1 and approximately 80% sensitivity, indicating that they cannot detect other serogroups [3]. The LAMP method has been useful in diagnosing Legionnaires’ disease in patients with negative urine antigens and sputum culture results for *L. pneumophila*, as observed in this case [3].

The patient presented with neurological symptoms at the onset of Legionnaires’ disease, including lower limb paresthesia, impaired consciousness, and frequent convulsive seizures. Retrospective analysis shows that 10% of cases of Legionnaires’ disease involve neurological symptoms, such as encephalitis, meningitis, peripheral nerve neuropathy, and brainstem abnormalities. Neurological symptoms were observed in 33.3% of patients who succumbed to Legionnaires’ disease [4]. The neurological symptoms of Legionnaires’ disease may also include psychiatric symptoms, such as confusion, hallucinations, and affective disorders, and cerebellar symptoms, such as gait disturbances, limb ataxia, and dysarthria [2]. Parkinsonism [2] and acute disseminated encephalomyelitis [5] have also been reported as neurological dysfunctions associated with Legionnaires’ disease. Seizures are also a neurological symptom of Legionnaires’ disease and occur in 1.5% of patients with Legionnaires’ disease with nervous system involvement [2]. A case of epileptic seizures after the treatment of *L. pneumophila* with moxifloxacin has been reported [6].

ASS is closely related to acute central nervous system damage caused by metabolic, toxic, structural, infectious, or inflammatory factors. ASS arises from various causes that alter central nervous system excitability and transiently lower seizure thresholds. Unlike epilepsy, ASS generally does not recur when the underlying cause is treated [7]. Frequent convulsive seizures during hospitalization were treated as ASS associated with systemic deterioration caused by Legionnaires’ disease and the use of antibiotics. The seizures improved following the administration of fosphenytoin and levetiracetam, as well as the discontinuation of antibiotic therapy.

The 10-year risk of unprovoked seizures following ASS is reported to be 18.7% [7]. In this case, the patient experienced episodes of impaired consciousness, automatism, and clouded awareness after discontinuing antiepileptic drugs, which were suggestive of epileptic seizures. Patients receiving antiepileptic drugs for severe ASS or epileptiform EEG may reduce their seizure risk by tapering the antiepileptic dose after confirming the absence of epileptiform discharges on EEG two to three-3 months after discharge [7]. Since benzodiazepines and levetiracetam may mask spike discharges, EEG testing after discontinuation has been suggested and should have been considered in this case [7].

Neuropsychological examination at the Medical Center for Dementia revealed mild cognitive impairment, while the WAIS-IV and Japanese Adult Reading Test indicated a decline in intellectual function. Previous research has shown that *L. pneumophila* infection impairs learning and memory in mice, with anti-interleukin (IL)-1 neutralizing antibodies improving task performance in infected mice [8]. IL-18, a marker of neuronal dysfunction, is elevated in cerebrospinal fluid in neurodegenerative conditions like Alzheimer’s disease and neuroinfectious diseases such as bacterial meningitis and human immunodeficiency virus-related dementia [9]. Elevated IL-1β and IL-18 levels were found in the brains of mice with *L. pneumophila*-induced pneumonia. Intracellular *L. pneumophila* infection induces primary microglial cells to release IL-1β and IL-18 and undergo pyroptotic programmed cell death [10]. Microglial cells may be activated during systemic infection, despite an intact blood-brain barrier. This suggests a flagellin-dependent neuroinflammatory process from peripheral inflammation due to Legionnaires’ disease [10]. Such mechanisms may have occurred in this case.

Hippocampal sclerosis is a primary cause of temporal lobe epilepsy, accounting for 56-70% of cases of refractory epilepsy in adolescents and adults that present as drug-resistant conditions [11]. Hippocampal sclerosis is characterized by reduced hippocampal volume and structural abnormalities, which may show T2/fluid-attenuated inversion recovery hyperintensity, gliosis, a dilated temporal horn, and blurred gray matter [12]. The cause of hippocampal sclerosis is unknown, but it has been associated with febrile convulsions during early childhood [13]. Cases of hippocampal sclerosis have also been reported following cerebral malaria, with MRI findings showing petechial microhemorrhage foci throughout the deep white matter and restricted diffusion in the hippocampus [14]. Similar cases have been observed after herpes simplex and varicella-zoster virus encephalitis [15]. These findings suggest that brain inflammation related to infection may play a role.

For this case, the clinical course and imaging findings showed no initial evidence of hippocampal atrophy during ASS during the acute phase of *Legionella *infection. However, memory impairment was detected four months later, along with MRI findings indicative of left hippocampal atrophy. Six months later, on the patient’s visit to our hospital, an MRI revealed left hippocampal sclerosis. The clinical course suggests that hippocampal sclerosis developed following *Legionella* infection. Hippocampal sclerosis may have been induced by *Legionella *infection itself, ASS, and hypoxic conditions.

This report has several limitations. Cerebrospinal fluid analysis was not performed during the acute phase of Legionnaires’ disease, leaving no definitive evidence of encephalitis. Additionally, the sole use of CT for imaging during ASS, and the initial MRI protocol was not specific for hippocampal assessment, making it impossible to completely rule out preexisting hippocampal sclerosis. However, the absence of any prior episodes of memory impairment or convulsions indicating temporal lobe epilepsy suggests that hippocampal sclerosis likely developed following the *Legionella *infection.

## Conclusions

This is the first reported case of hippocampal sclerosis occurring several months after the onset of *Legionella *infection. It demonstrates the potential for residual risks of cognitive impairment, epileptic seizures, and altered consciousness in patients recovering from Legionnaires’ disease. Therefore, clinicians should perform careful follow-up assessments. The decision to discontinue antiepileptic medications should be made with caution when seizures are induced by Legionnaires’ disease because of the risk of recurrence. Further reports of similar cases are anticipated to contribute to a deeper understanding of this rare condition.
